# On the Benefit from Cold Applied to the Head in the Fever Called Typhus

**Published:** 1817-10

**Authors:** J. Wood


*For the London Medical and Physical Journal.*

### *On the Benefit from Cold applied to the Head in the Fever called Typhus;* by J. Wood, M.D.

WHEN I consider how much good may be done by making known a single fact through the medium of a public journal, a fact simple and efficient in its operation, and applicable to a disease frequent in this and other countries, which may be communicated in that manner with a quickness not to be exceeded, I am induced to attempt to effect that good by the following communication. If the disease is universal, if the fact is universally applicable, and if the journal is circulated (as I believe the London Medical and Physical Journal is) to all points in this and to many other countries, through Europe, in America, and in the Indies, it is impossible to calculate the good that may be effected by the small labour of writing a few lines.

For

For above twenty years I have applied cold to the head with uniform good effects, in all cases of insanity from too high action of the brain, which is almost always attended with redness of the vessels of the tunica conjunctiva of the eye, and a strong and quickened pulse. I have also used it* as successfully in that state of the brain called brain-fever, peculiar to persons taking spirits to excess, during the same period; but it is only within a few years that I have used it in typhus fever, which has prevailed much here, and in all parts, I believe, of this country, for some months past. I have seldom applied it at an early period of the fever, but have rather waited for the necessity of it, and I have never been disappointed: it soon abates the most painful restlessness and pain in the head, and procures sleep, and this too at a time when no other power can do it, at a time when the physician feels almost at a loss for further means, when the patient is highly restless, and often uttering indications of pain, and when the friends are in a state of great anxiety and alarm. When the cold is applied very effectually, the skin, so long hot, and obstinately dry, generally opens with considerable perspiration. It is not only from my own experience that I advance what I say, but also on that of others, whose reports to me are uniform in every particular of its pleasant and decided effects.

I now proceed to the mode of applying the cold to the head. It has always been done by large towels, dipped in the coldest water, and immediately laid all over the head, face, and back of the neck, one after another: as soon as one has been on half a minute, it is removed, and another must be instantly applied, and thus continued in as quick succession for ten or fifteen minutes, until it is seen that the patient is easier, the heat less, and a disposition to sleep has taken place. It is generally necessary to repeat the operation at first at short intervals, and it is very desirable to do it with so much quickness and perseverance as to produce *some degree of shivering*,† the effects being more permanent when this has taken place. The feet are sometimes cold when the rest of the body is hot, and this is often the case in typhus; and I therefore always used means to warm the feet when the cold water was applied to the head, and often had the *feet* in *warm water* while the *cold water* was so applied. I had found warm water to the feet, and cold water to the head, useful in mania, and sometimes in epileptic fits,

* As mentioned in the 19th vol. of this Journal, p. 231 and 321.

† I have always found this quite necessary in brain-fever, as relief was never obtained unless this effect had been produced.

in in which so little can be done; and, from analogy, I concluded the same might be useful in typhus, when there seemed great excitement in the brain, with heat, want of sleep, great restlessness, and sometimes delirium, and I have fully succeeded. In a few instances of typhus, when recovery seemed hopeless in the evening, the patients have been declared the following morning to be out of danger, after the application of cold water to the head and warm water to the feet, as above mentioned.

I might have observed how particularly desirable such means of relief are for children, with whom there is often a difficulty in giving medicine—means that afford a complete power,—general, quick, and decided.

*Newcastle; August* 20, 1817.

---

*For the London Medical and Physical Journal.*

*Case of obstinate Constipation;* by R. H. KING, Esq.

